# Neurogenesis in directly and indirectly developing enteropneusts: of nets and cords

**DOI:** 10.1007/s13127-015-0201-2

**Published:** 2015-01-31

**Authors:** Sabrina Kaul-Strehlow, Makoto Urata, Takuya Minokawa, Thomas Stach, Andreas Wanninger

**Affiliations:** 1 Department of Integrative Zoology, University of Vienna, Althanstr. 14, 1090 Vienna, Austria; 2 Takehara Marine Science Station, Setouchi Field Science Center, Graduate School of Biosphere Science, Hiroshima University, 5-8-1 Minato-machi, Takehara, Hiroshima 725-0024 Japan; 3 Research Center for Marine Biology, Tohoku University, Asamushi, Aomori, Aomori 039-3501 Japan; 4 Institute for Biology, Humboldt-University Berlin, Philippstr. 13, 10115 Berlin, Germany

**Keywords:** Nervous system, Evolution, Enteropneusts, Hemichordates, Deuterostome, Serotonin, Neurogenesis, Plexus, Bipolar receptor cell

## Abstract

Concerning the evolution of deuterostomes, enteropneusts (acorn worms) occupy a pivotal role as they share some characteristics with chordates (e.g., tunicates and vertebrates) but are also closely related to echinoderms (e.g., sea urchin). The nervous system in particular can be a highly informative organ system for evolutionary inferences, and advances in fluorescent microscopy have revealed overwhelming data sets on neurogenesis in various clades. However, immunocytochemical descriptions of neurogenesis of juvenile enteropneusts are particularly scarce, impeding the reconstruction of nervous system evolution in this group. We followed morphogenesis of the nervous system in two enteropneust species, one with direct (*Saccoglossus kowalevskii*) and the other with indirect development (*Balanoglossus misakiensis*), using an antibody against serotonin and electron microscopy. We found that all serotonin-like immunoreactive (LIR) neurons in both species are bipolar ciliary neurons that are intercalated between other epidermal cells. Unlike the tornaria larva of *B. misakiensis*, the embryonic nervous system of *S. kowalevskii* lacks serotonin-LIR neurons in the apical region as well as an opisthotroch neurite ring. Comparative analysis of both species shows that the projections of the serotonin-LIR somata initially form a basiepidermal plexus throughout the body that disappears within the trunk region soon after settlement before the concentrated dorsal and ventral neurite bundles emerge. Our data reveal a highly conserved mode of neurogenesis in enteropneusts that is independent of the developing mode and is inferred to be a common feature for Enteropneusta. Moreover, all detected serotonin-LIR neurons are presumably receptor cells, and the absence of serotonin-LIR interneurons from the enteropneust nervous system, which are otherwise common in various bilaterian central nervous systems, is interpreted as a loss that might have occurred already in the last common ancestor of Ambulacraria.

## Introduction

**Fig. 1:**
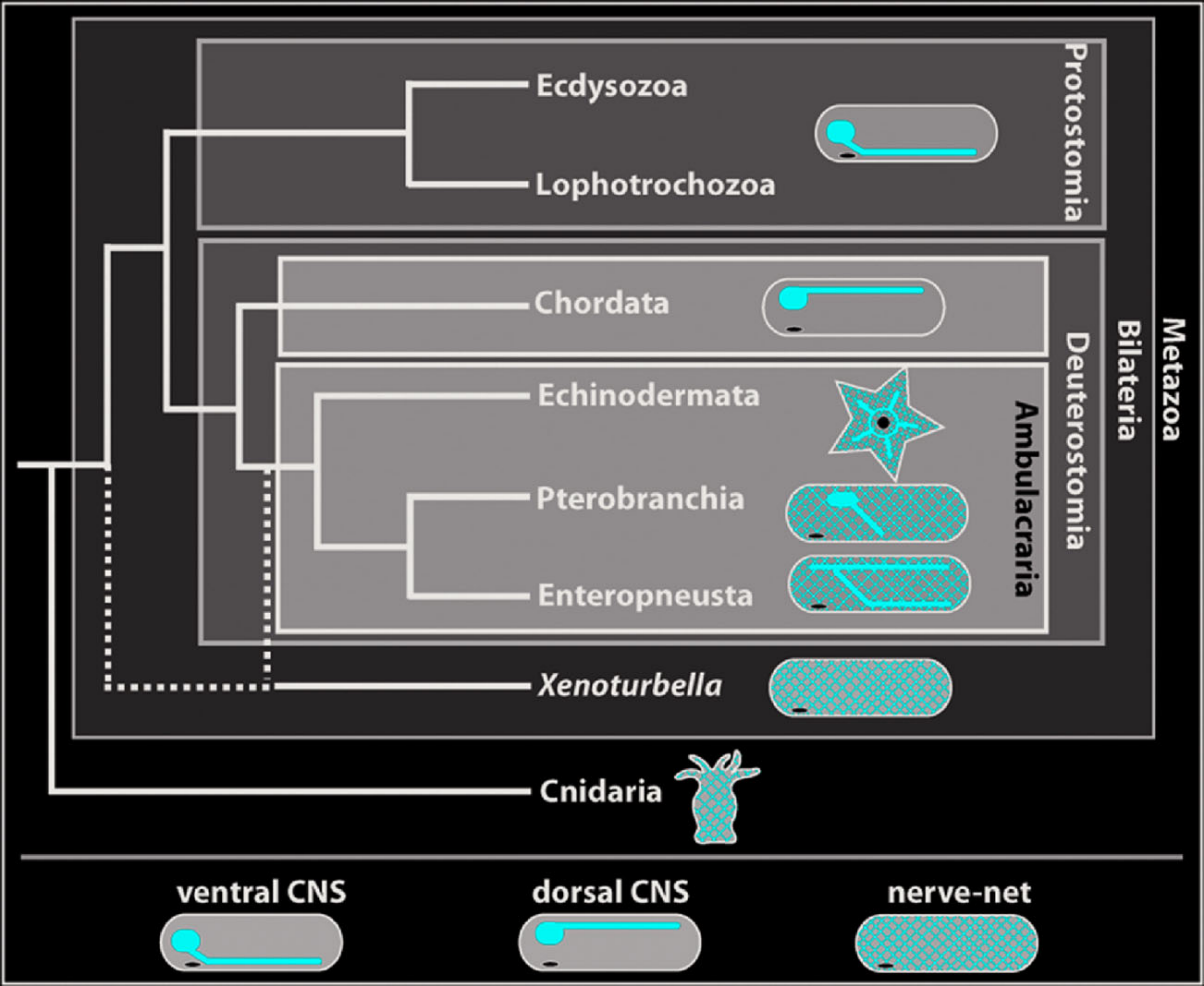
Phylogenetic relationships of major metazoan taxa compiled from recent molecular analyses (i.e., Hejnol et al. 2009; Philippe et al. 2011). Schematic representation of the adult nervous system is included for each taxon. For more information, see text

Numerous comparative studies on the nervous system have revealed highly variable neural anatomies in multicellular animals, ranging from a simple nerve net in cnidarians to highly complex central nervous systems (CNS) in vertebrates (Nieuwenhuys et al. 1998) and some protostomes (e.g., arthropods and cephalopods) (Bullock and Horridge 1965; Nixon and Young 2003; Strausfeld 2012). In Ambulacraria, a group of nonchordate deuterostomes that include echinoderms, pterobranchs, enteropneusts, and arguably xenacoelomorphs (Philippe et al. 2011), the nervous system of most of them comprises both a basiepidermal plexus as well as centralized portions (Fig. 1) (Bullock 1946; Cavey and Märkel 1994; Chia and Koss 1994; Knight-Jones 1952; Stach et al. 2012). It is still debated whether a plexus-like nervous system or a concentrated CNS was present in the last common ancestor of deuterostomes (LCAD). On the one hand, molecular analyses of the enteropneust *Saccoglossus kowalevskii* show circumferential expression of neuro-patterning genes throughout the entire ectoderm (Lowe et al. 2003; Pani et al. 2012; Cunningham and Casey 2014), thus supporting the presence of a basiepidermal plexus in the LCAD (Holland 2003; Gerhart et al. 2005). On the other hand, similarities in the anteroposterior and mediolateral patterning including presence and position of specific neuronal types between the ventral CNS of some protostomes (annelids and arthropods) and the dorsal CNS of chordates lead some authors to propose a CNS in the LCAD (Denes et al. 2007; Arendt et al. 2008; Nomaksteinsky et al. 2009; Holland et al. 2013; Miyamoto and Wada 2013). Both contradicting hypotheses are mainly based on molecular genetic analyses, whereas potentially informative neuro-anatomical data has barely been integrated. And, this is because data on neural morphogenesis in juvenile enteropneusts still remain scarce (e.g., Kaul and Stach 2010; Miyamoto et al. 2010) and precise anatomical descriptions of the nervous system are mainly based on histological analyses of adult specimens (Bullock 1946; Knight-Jones 1952).

During the last decade, the broad usage of antibodies against neuroactive substances such as serotonin, RFamides, and others in combination with immunofluorescent dyes has been proven highly successful and has fuelled studies on neurogenesis and neuroanatomical descriptions on various multicellular animals (Hay-Schmidt 2000; Harzsch et al. 2009; Wanninger 2009; Fritsch and Richter 2010; Miyamoto et al. 2010; Richter et al. 2010; Stach et al. 2012). In particular, serotonin (or 5-hydroxytryptamine, 5-HT), a biogenic amine that functions as a neurotransmitter, is distributed throughout all animal phyla and has a long evolutionary history (see, e.g., Osborne 1982, Fujii and Takeda 1988; Hay-Schmidt 2000). Neurogenesis in enteropneusts has been documented only in single or few developmental larval stages of indirect developers (Dautov and Nezlin 1992; Hay-Schmidt 2000; Nakajima et al. 2004; Nezlin and Yushin 2004). The two studies dealing with metamorphosis in enteropneusts (Nielsen and Hay-Schmidt 2007; Miyamoto et al. 2010) allow only an overview on the developing adult nervous system; yet, no details of, e.g., the collar cord, the nerve cords, or individual neurons have been addressed. Moreover, immunocytochemical data on neurogenesis in directly developing enteropneusts such as *Saccoglossus* sp. are entirely lacking. Such comparative data are, however, an important prerequisite for any inferences concerning nervous system evolution and for understanding the structure of the nervous system in the ground pattern of Enteropneusta and eventually Ambulacraria.

In order to contribute to such a comparative database, we investigated neurogenesis in *Balanoglossus misakiensis*, an indirectly developing enteropneust, as well as in *S. kowalevskii*, a direct developer, on a cytological and ultrastructural level from early metamorphosis through juvenile life. Hereby, we provide the first detailed comparative analysis in two enteropneust species which contributes to our understanding of the ground pattern of Enteropneusta.

## Materials and methods

### *Balanoglossus misakiensis* (Kuwano, 1902)

Adult *B. misakiensis* were collected at a depth of 1 to 2 m at Sunset beach, Aomori-Bay, Asamushi, Aomori, Japan, in June 2012. Specimens were maintained in aquaria with running seawater at ambient water temperature (24–26 °C) at the Research Center for Marine Biology Tohoku University in Asamushi. Spawning of gravid females was induced by elevating the temperature to 31 °C, and insemination was carried out artificially (Urata and Yamaguchi 2004). Embryos were cultured in membrane-filtered seawater in an incubator at 24 °C in Petri dishes without agitation. Hatched larvae were transferred into 5-l beakers at a concentration of 2 larvae/10 ml and were cultured with gentle stirring. From 2-day postfertilization (pf) onward, they were daily fed a mixture of the single-celled algae *Chaetoceros gracilis*, *Dunaliella* sp., and *Pavlova luteri*. Most of the water in the beaker was replaced by fresh membrane-filtered seawater every day, and streptomycin (50 mg/l) was added to prevent fungal growth. Agassiz-stage larvae (14 days pf) were cultured with sand grains from their natural habitat in Petri dishes to induce metamorphosis.

### *Saccoglossus kowalevskii* (Agassiz, 1873)

Adult specimens of *S. kowalevskii* were collected from intertidal flats near Woods Hole (Massachusetts, USA) in September 2007 and 2011. Animals were transported into the laboratory, separated according to sex, and kept individually in finger bowls. Animals were kept at 18 °C on a seawater table. Freshly spawned eggs were mixed with active sperm isolated from a ripe male and diluted in seawater (Lowe et al. 2004). Fertilization envelopes were ruptured using fine forceps, and embryonic and juvenile stages were collected using Pasteur pipettes. Embryos were relaxed in a mix of 7 % MgCl_2_ and seawater (1:1) for 5–10 min prior to fixation for transmission electron microscopy (TEM) and scanning electron microscopy (SEM).

### Terminology of names of developmental stages

For indirectly developing enteropneusts such as *B. misakiensis*, we follow the terminology sensu Nielsen and Hay-Schmidt (2007). Accordingly, the Spengel stage is characterized by a regression of size of the larva and the fusion of the preoral ciliary feeding band, the neotroch. The Spengel stage is followed by the Agassiz stage. Agassiz larvae have a cone-shaped anterior body region with a smooth surface, the prospective proboscis region. The overall size is further decreased, and the anterior eye spots begin to disappear. The Agassiz larvae are competent for settlement.

For the direct developer *S. kowalevskii*, we followed the terminology of Lowe et al. (2003, 2006). Accordingly, the Kink stage is characterized by a dorsal bending of the future trunk region.

### Electron microscopy

TEM and SEM embryos and juveniles of *S. kowalevskii* were fixed with ice-cold 2.5 % glutaraldehyde (GA) in 0.2 M sodium cacodylate buffer (pH 7.2), adjusted to an osmolarity of approximately 800 mosm with the addition of NaCl (for *B. misakiensis*, 2.5 % GA in 0.05 M phosphate buffer + 0.3 M sodium chloride (PBS)). Primary fixation was stopped after 45 min (30 min, *B. misakiensis*) with three buffer rinses of 10, 15, and 20 min, respectively (five rinses with PBS for 10 min each for *B. misakiensis*). Primary fixation was followed by 30 min of postfixation with 2 % OsO_4_ in sodium cacodylate buffer (PBS). Postfixation was stopped with three buffer rinses (15, 30, 30 min) followed by two rinses with ddH2O (15, 30 min). After dehydration through a graded series of ethanol, specimens were embedded in Epon resin for TEM and light microscopy. For SEM, specimens were critical point dried in a CPD 030 (Balzers Union, Liechtenstein) or Leica CPD 300 (Leica, Germany). Dried specimens were sputter coated with gold in a SCD 040 (Balzers Union, Liechtenstein) sputter coater and viewed with a Fei Quantum 200 SEM at 15 kV (FEI Co., The Netherlands) or a Philips XL 30 ESEM (Philips, The Netherlands). Complete longitudinal and transverse semithin serial sections (0.5 μm) for light microscopy and ultrathin serial sections (~55 nm) for TEM from three stages of *S. kowalevskii* (56, 132, and 432 h pf) were produced on a Leica Ultracut S microtome and from five stages of *B. misakiensis* (“Spengel stage”: 13 days pf, “Agassiz stage”: 14 days pf, 12-h postsettlement (ps), 1 ay ps and 3 days ps) sectioned on a Leica UC 7 microtome. Semithin sections were stained with 1 % toluidin blue for 3 min at 63 °C. Ultrathin sections were stained with 2 % uranylacetate and 2.5 % lead citrate, either in an automatic stainer (NanoWlm Technologie GmbH, Göttingen, Germany) or manually. Light microscopic images were recorded with a digital camera (Olympus BX-UCB) mounted on an Olympus BX51 compound microscope or Nikon Eclipse E800. TEM pictures were documented with a Philips EM 208 electron microscope at 70 kV equipped with a Nikon digital camera. Images were optimized by using Adobe Photoshop and Illustrator CS3 (Adobe, San José, CA, USA).

### Immunolabeling and confocal laser scanning microscopy 

For immunocytochemistry embryos and juveniles of *S. kowalevskii* (dorsal kink = 76 hpf, 1-gill-slit hatchling = 156 hpf, 3-gill-slit juvenile = 750 hpf) and larvae and juveniles of *B. misakiensis* (“Spengel stage” = 13 days pf, “Agassiz stage” = 14 days pf, early settled stage = 12-h postsettlement (ps), 1-gill-slit juvenile = 1 day ps, and 2-gill-slit juvenile = 3 days ps) were fixed with 4 % paraformaldehyde (PFA) in phosphate buffer (PB). Specimens were washed three times and permeabilized in PB containing 2 % Triton X-100 (PBT). Subsequent blocking was carried out in PBT containing 6 % normal goat serum (Jackson Immuno Research) for 2 h at room temperature (RT). Primary antibodies were diluted in blocking solution in PBT and applied at a final concentration of 1:500 (anti-serotonin, Sigma, Cat. # S5545; anti-acetylated α-tubulin Sigma, Cat. # T6793) overnight at RT. Then, animals were washed six times for 20 min each with PBT on a rocker table. Secondary antibodies were diluted in blocking solution in PBT and applied at a final concentration of 1:600 (goat anti-rabbit Alexa Fluor 633, Invitrogen, Cat. # A21070; goat anti-mouse Alexa Fluor 568, Invitrogen, Cat. # A11004) together with a nuclei marker (1:600, DAPI, Invitrogen, Cat. # D1306) and a marker for filamentous actin (1:150, Alexa fluor 488 phalloidin, Invitrogen, Cat. # A12379) for 4 h at RT. Afterward, the samples were rinsed three times for 20 min each in PBT and twice in PBS. Subsequently, samples were stepped gradually into 70 % glycerol and eventually mounted in Fluoromount G (SouthernBiotech) on glass slides. The strong signal resulting from the epidermal cilia and mesodermal muscles made it impossible to unambiguously identify neural structures such as neurites in the tubulin and phalloidin stainings; yet, the detected cilia and muscles were used as positional markers. Analysis and digital image acquisition were performed on a Leica TCS SP5 II confocal laser scanning microscope (Leica Microsystems, Germany). Optical sections were taken at a step size between 0.3 and 0.6 μm in Z-resolution. The resulting image stacks were digitally merged into maximum Z-projections and further processed with the open source image software Fiji (Max Planck Society for the Advancement of Science e.V., Germany), Adobe Photoshop, and Illustrator CS3 (San José, CA, USA). At least ten specimens per stage were stained and scanned and rendered identical results for each staining per stage.

Negative controls were performed by excluding either the primary or the secondary antibody. No signal was detected in any specimens of these experiments. In order to test for unspecific binding of the anti-serotonin (5-HT) antibody, additional negative controls with preadsorbed antibodies were performed on developmental stages of *B. misakiensis* and *S. kowalevskii*. For this, the rabbit anti-serotonin (5-HT) antibody (polyclonal; ImmunoStar) was incubated in PBT blocking solution overnight at 4 °C together with serotonin (5-HT-)-BSA conjugate (ImmunoStar) reconstituted in PBT block with a final dilution of the antibody of 1:500 and a final concentration of the serotonin-BSA conjugate of 20 μg/ml. This solution was subsequently used as primary antibody solution according to the protocol described above, and none of the individuals showed any signal.

## Results

### *Balanoglossus misakiensis*: Spengel stage

**Fig. 2:**
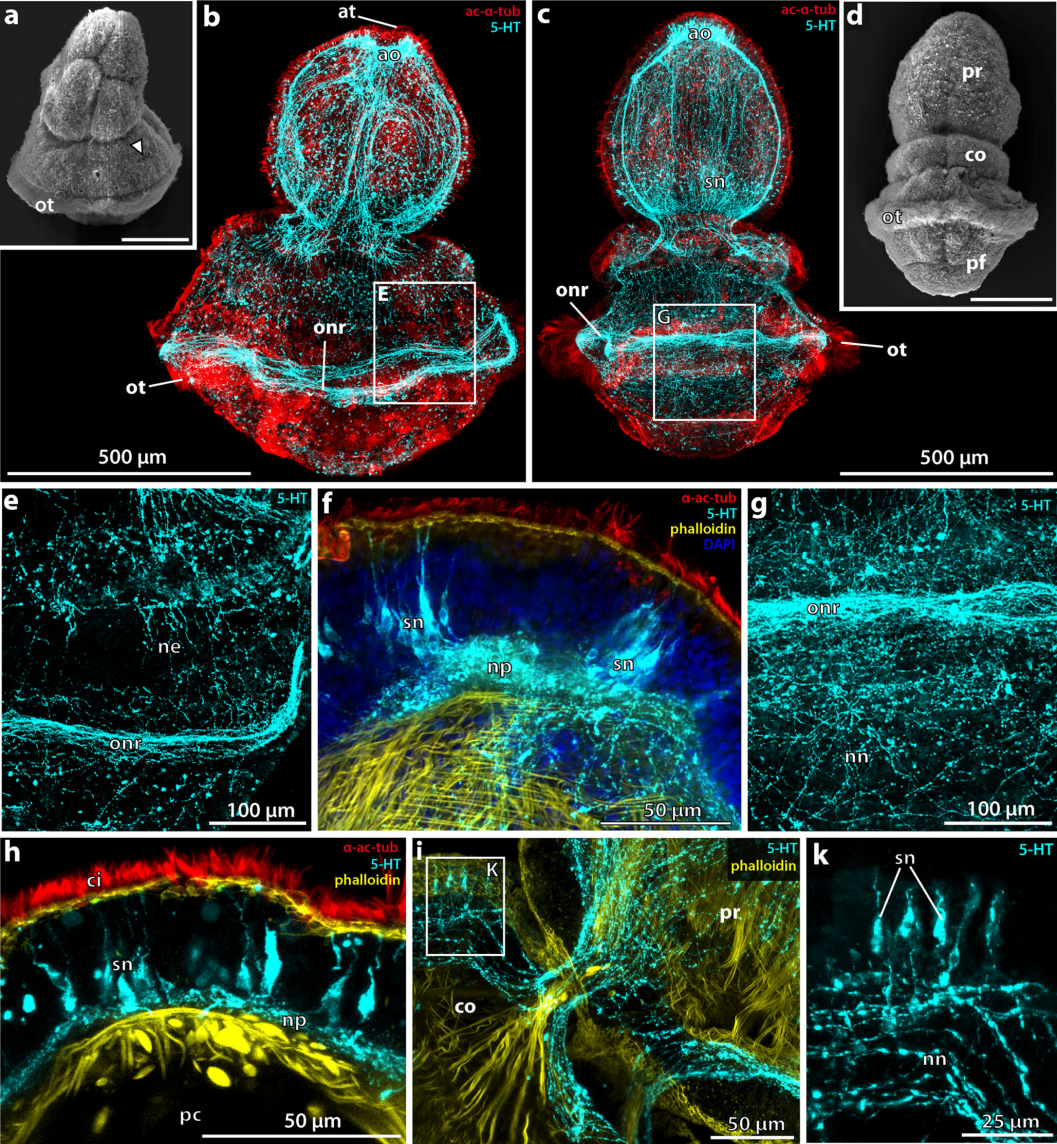
Neurogenesis in metamorphosing stages of *B. misakiensis*. **b**, **c**, **e**–**k** Z-projections of confocal microscopy image stacks. **a**, **d** Scanning electron micrographs. Anterior is to the *top*. **a** SEM of early metamorphosing stage (Spengel) from lateral left. *Arrowhead* points to the dorsolateral slit-like depression. **b** Overview of the serotonin-LIR nervous system (NS) in Spengel stage, view from *left.* Numerous serotonin-LIR neurons are part of the apical organ. Note the serotonin-LIR opisthotroch neurite ring. **c** Overview of the serotonin-LIR NS of a late metamorphosing stage (Agassiz), dorsal view. **d** Dorsal view of metamorphosing Agassiz stage. **e** Detail showing the developing 5-HT+ nervous plexus in the postoral region of the Spengel stage. **f** Close-up of the 5-HT+ apical organ of the Spengel stage in lateral view. Note the anterior and posterior cluster of bipolar neurons connected by a central neuropil. **g** Detail showing the elaborated 5-HT+ nervous plexus in the prospective trunk region of an Agassiz stage larva. **h** Detail view of 5-HT+ apical bipolar neurons. A slender distal neurite connects the soma to the apical cell surface. **i** Detail of the neuite bundles passing the neck region, connecting the proboscis to the collar region. **k** Close-up of the dorsal collar region showing the 5-HT+ bipolar neurons. 5-HT, serotonin; ac-α-tub, acetylated α-tubulin; ao, apical organ; at, apical tuft; ci, cilia; co, collar; ne, neurite; nn, nervous plexus; np, neuropil; onr, opisthotroch nerve ring; ot, opisthotroch; pf, perianal field; pr, proboscis; sn, serotonin-LIR neuron; tr, trunk

After about 13 days postfertilization (pf), the tornaria larvae start transforming into the Spengel stage accompanied by a series of morphological changes. The larvae are approximately 950 μm in length and are subdivided by a deep circular constriction into a slightly conical preoral region forming the future proboscis and a voluminous spherical posterior region bearing the distinct opisthotroch (Fig. 2a, b). The oral field is almost completely obliterated as the ciliary bands of the neotroch are fused together. The position of the ciliary bands is still indicated by grooves (Fig. 2a). A pair of dorsolateral slit-like depressions on the anterior part of the postoral body indicates the position of the prospective first gill pores (Fig. 2a, arrowhead).

The entire larva is covered by short cilia. An apical tuft composed of longer cilia is present at the apical tip (Fig. 2b). Within the apical epidermis, numerous serotonin-LIR neurons are grouped together to form an anterior and a posterior cluster of cells which are interconnected by a median neuropil (Fig. 2f). The serotonin-LIR somata are slender, sometimes flask-shaped, cells with a nucleus that is positioned centrally or basally within the cytoplasm. Each soma is connected to the apical cell surface by a narrow elongation, the distal neurite (Fig. 2f). The serotonin-LIR cells are bipolar neurons that send a neurite into the median neuropil of the apical organ. Aside from the cells of the apical organ, numerous serotonin-LIR somata are scattered throughout the proboscis’ epidermis. Each bipolar neuron exhibits a distal neurite connecting to the apical surface and a proximal neurite. The proximal neurites of those bipolar neurons form a basiepidermal plexus-like arrangement throughout the proboscis epidermis. Within the voluminous postoral region, serotonin-LIR somata are only occasionally present. A conspicuous number of neurites are present at the level of the opisthotroch and form a distinct neurite bundle, namely, the opisthotroch neurite ring (Fig. 2b, e). Aside from that neurite bundle, the postoral body shows only individual neurites arranged in a loose, sometimes discontinuous pattern (Fig. 2e).

### *B. misakiensis*: Agassiz stage

The larvae of the Agassiz stage (14 days pf) are of elongated shape measuring approximately 1 mm in length and 650 μm in width. The anterior proboscis is of conical acorn shape (Fig. 2c, d). It is separated from the posterior body by a deep circular constriction (Fig. 2c, d). The grooves of the fused ciliary bands are no longer visible externally (compare Fig. 2a and d). A second circular constriction indicates the posterior margin of the collar region (Fig. 2d). The collar region is about 150 μm long. The remaining posterior part of the body constitutes the future trunk region. It measures approximately 400 μm in total length and bears the opisthotroch at the midlevel of the trunk region (Fig. 2c, d).

**Fig. 3:**
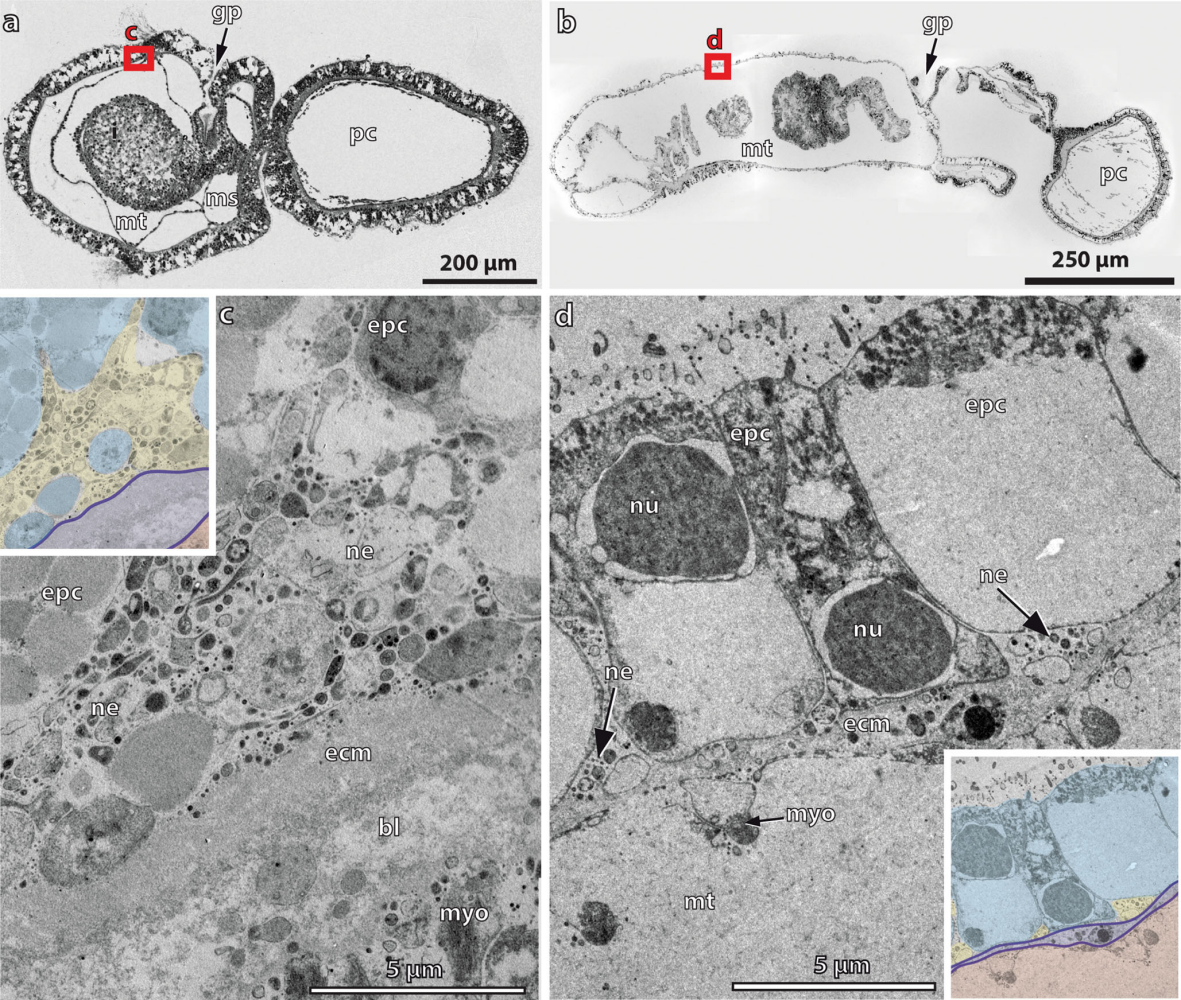
Ultrastructural details of the developing nervous system *Balanoglossus misakiensis*. **a** Sagittal section of an Agassiz stage larva of *B. misakiensis*. **b** Sagittal section of a 2-gill-slit juvenile of *B. misakiensis.* **c** Ultrastructural detail of the red-marked box in **a** showing a continuous layer of neurites of 4–6-μm thickness in the Agassiz stage. *Inset* color-coded image of the micrograph shown in **c. d** Ultrastructural detail of the red-marked box in **b** showing only individual basiepidermal neurites in the juvenile. *Inset* Color-coded image of the micrograph shown in **d**. Color code for insets: ectoderm, *light blue*; neurites, *yellow*; basal lamina (ecm), *dark blue*; mesoderm, *red*. bl, blastocoel; ecm, extra cellular matrix; epc, epidermal cell; gp, gill pore; i, intestine; ms, mesocoel; mt, metacoel; mtc, metacoelic cell; myo, myofilaments; ne, neurite; nu, nucleus; pc, protocoel; yo, yolk

Serotonin-LIR is present at the anterior tip of the proboscis region (Fig. 2c) and reveals numerous bipolar neurons with thin and long extensions to the cell surface where each neuron bears a single cilium (Fig. 2h). The overall morphology of the serotonin-LIR bipolar neurons of the apical organ is comparable to that of the Spengel stage. Further individual serotonin-LIR neurons are interspersed throughout the proboscis with a higher density present near the base of the proboscis (Fig. 2c, sn). All serotonin-LIR somata project into a basiepidermal plexus that is present throughout the proboscis. The plexus of the proboscis region is connected to that of the collar region by two laterally condensed neurite bundles that pass through a neck-like transition into the collar region (Fig. 2c, i). A pair of bilateral muscle bundles that run anteriorly into the base of the proboscis accompanies the neurite bundles (Fig. 2i). Several serotonin-LIR bipolar neurons are present within the anterior collar region (Fig. 2i, k), whereas only scattered neurons are present within the prospective trunk region. The number of neurites within the collar and trunk region has considerably increased compared to the Spengel stage, and the neurites show a distinct plexus-like pattern, particularly in the prospective trunk region (Fig. 2g). The opisthotroch nerve ring is still well developed and encircles the larva’s body in the middle of the prospective trunk region (Fig. 2c). Ultrastructural analyses of the metamorphosing Agassiz stage of *B. misakiensis* revealed the presence of numerous neurites that form a 4–6-μm-thick basiepidermal layer within the prospective trunk region (Fig. 3a, c, inset).

### *B. misakiensis*: early settled juvenile

**Fig. 4:**
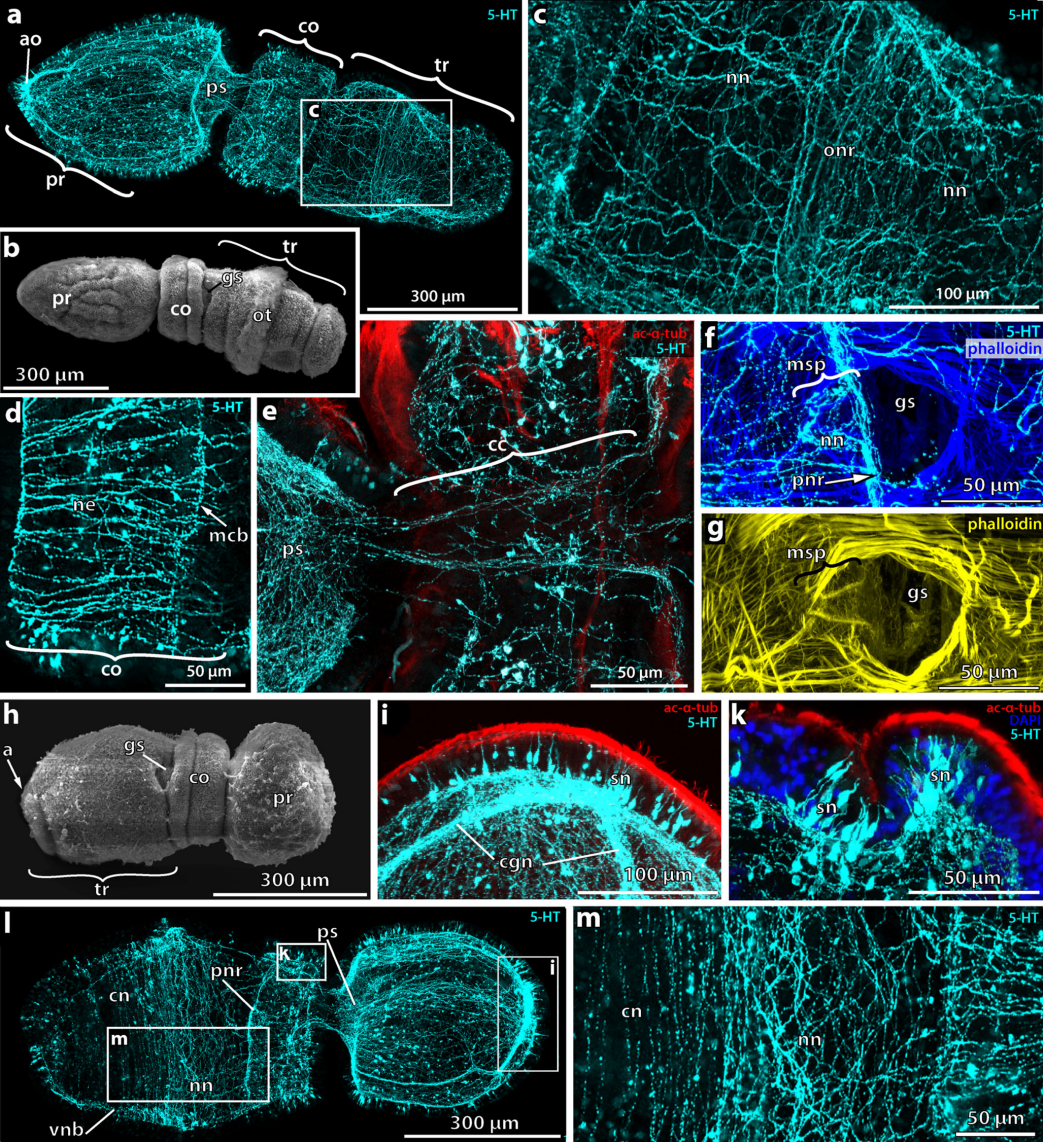
Neurogenesis in early settled stages of *B. misakiensis*. **a**, **c**–**i**, **l**, **m** Z-projections of confocal microscopy image stacks. **b**, **k** Scanning electron micrographs. **a** Ventrolateral overview of the serotonin-LIR NS of an early settled juvenile. Anterior is to the left. **b** Left side view of early settled juvenile. **c** Detail of the anterior trunk region of early settled juvenile showing a ubiquitous basiepidermal nervous plexus. **d** Partial Z-projection showing the longitudinally orientated neurites within the collar region. **e** Detail of the 5-HT+ proboscis plexus at the base of the proboscis and the neurite bundles passing through the subepidermal collar cord. **f**, **g** Close-up of the gill pore and mesocoelic pore showing the prebranchial nerve ring and the nervous plexus entangling the mesocoelic duct in **f. h** SEM of a settled juvenile in lateral right view. **i** Close-up of the apical tip of the proboscis. **k** Detail of the dorsolateral collar region showing two clusters of 5-HT+ bipolar neurons that encircle the collar. **l** Overview of the 5-HT+ NS of a settled juvenile 24-h postsettlement, lateral right view. **m** Detail showing circular 5-HT+ neurites within the posterior part of the trunk whereas more anterior a net-like pattern is still present. The apical tuft and cluster of 5-HT+ somata has disappeared. Apical is to the top. 5-HT, serotonin; ac-α-tub, acetylated α-tubulin; a, anus; ao, apical organ; cgn, ciliary groove nerves; cn, circumferential neurite; cc, collar cord; co, collar; gs, gill slit; mcb, middle circular neurite bundle; msp, mesocoel pore; ne, neurite; nn, nervous plexus; onr, opisthotroch nerve ring; pnr, prebranchial nerve ring; pr, proboscis; ps, proboscis stem; sn, serotonin-LIR neuron; tr, trunk; vnb, ventral neurite bundle

Early settled *Balanoglossus* (12-h postsettlement (ps)) have an elongated body of vermiform shape measuring approximately 1 mm in length (Fig. 4a, b). The proboscis is pointed at the anterior tip and about 450 μm long. The collar region measures 200 μm in length and is subdivided by a circular constriction into a broader anterior and narrower posterior part. The trunk region has significantly increased in length, now being 430 μm long. One pair of dorsolateral gill pores is developed at the anterior margin just behind the collar region. The opisthotroch is still present and encircles the trunk region (Fig. 4b).

Numerous serotonin-LIR somata are distributed throughout the epidermis of the proboscis region, and the apical organ is still visible at the anterior tip; yet, the number of serotonin-LIR cells is reduced (Fig. 4a) compared to the Agassiz stage. The serotonin-LIR nervous system of the proboscis region differs from the Agassiz stage in having a neurite-rich plexus at the dorsomedian base of the proboscis (Fig. 4a, e). This so-called proboscis stem comprises one of the most condensed areas of the adult enteropneust nervous system. Posterior of the proboscis plexus, two serotonin-LIR neurite bundles project into the collar region and pass through the collar on a subepidermal level (Fig. 4e). Both serotonin-LIR bundles are part of the neurulated collar cord that forms at this stage. Although DAPI staining reveals numerous nuclei accompanying the collar cord, no soma stains positively for serotonin within the collar cord. At the posterior end of the collar, the two neurite bundles become basiepidermal again and project laterally (Fig. 4e) into the prebranchial nerve ring (see below). Within the epidermis of the collar region, numerous serotonin-LIR somata are intercalated between the epidermis cells. Each serotonin-LIR bipolar neuron sends a proximal neurite into the basiepidermal plexus. The majority of serotonin-LIR neurites within the collar region runs in longitudinal direction (Fig. 4d) and project into one of the two circular serotonin-LIR neurite bundles within the posterior half of the collar region (Fig. 4d, mcb and 4f, pnr). The more anterior circular neurite bundle runs basiepidermally at the level of the internal margin of the collar coelom (mesocoel) (Fig. 4d, mcb), whereas the more posterior ring, the prebranchial nerve ring, is present at the very posterior margin of the collar region. The prebranchial nerve ring forms a distinct network of serotonin-LIR neurites around the paired mesocoelic pores (Fig. 4f, g). These mesocoelic pores connect the coeloms of the collar region, the mesocoels, to the opening of the first gill pore, and eventually to the exterior. At this stage of development, a basiepidermal plexus is detectable by serotonin-LIR throughout the entire trunk region (Fig. 4c), however, with only few serotonin-LIR somata. The opisthotroch neurite ring is less condensed and comprises only a loose arrangement of circular neurites (Fig. 4a, c).

### *B. misakiensis*: 1-gill-slit juvenile

After approximately 1-day postsettlement, the majority of juveniles have lost the opisthotroch (Fig. 4h). The proboscis shape has slightly changed into a blunt, rounded acorn, measuring between 250 and 350 μm in length. The collar region resembles that described for the previous stage and the trunk region is shorter than the previous stage (Fig. 4h, l).

Within the proboscis and collar region, serotonin-LIR reveals only few changes of the overall architecture of the nervous system. Along the anterior tip of the proboscis, the number of the serotonin-LIR somata within the former apical organ is further reduced (Fig. 4i, l). Along the anterior-posterior axis of the proboscis region, distinct neurite bundles indicate the position of the former larval ciliary bands (Fig. 4i, cgn, l). Within the anterior two thirds of the collar region, two rings of numerous serotonin-LIR neurons are present (Fig. 4k). These cells are long, slender bipolar neurons with the somata placed basally, centrally, or even apically within the epidermis (Fig. 4k). At the posterior collar margin, a pair of prebranchial nerves runs circumferentially, passes the mesocoelic pores, and eventually connects to the ventral part of the nervous plexus (Fig. 4l, pnr). While the arrangement and orientation of the basiepidermal neurites within the proboscis and collar region have not changed much, the situation in the trunk region is considerably different. A plexus-like arrangement of serotonin-LIR neurites is only detectable in the anterior half of the trunk (Fig. 4l, m), whereas in the posterior part of the trunk, individual circumferential neurites are present. These circular neurites project into a longitudinal neurite bundle that is present along the ventral midline (Fig. 4l, vnb).

### *B. misakiensis*: 2-gill-slit juvenile

**Fig. 5:**
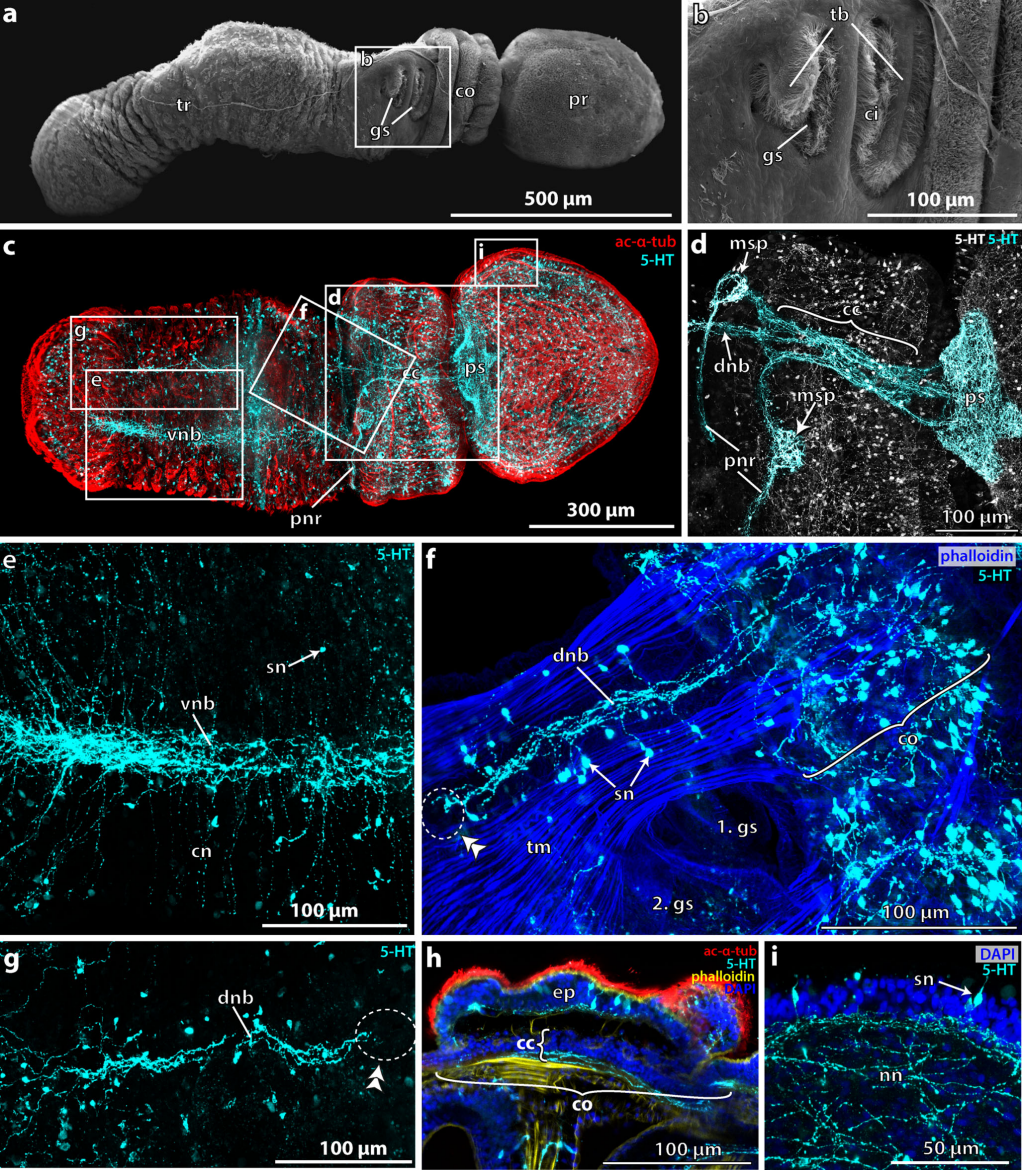
Neurogenesis in the 2-gill slit juvenile of *B. misakiensis*. **a, b** Scanning electron micrographs: **c–i** Z-projections of confocal microscopy image stacks. Anterior is to the right. **a** Dorsolateral view of a 3-day-old settled juvenile. **b** Higher magnification of the two gill slits and the dorsal tongue bars. **c** Overview of the 5-HT+ NS of a 3-day-old juvenile. **d** The collar cord is composed of three 5-HT+ neurite bundles of which the median one projects posteriorly into the dorsal nerve cord. **e** Detail of the ventral 5-HT+ neurite bundle with numerous incoming circular neurites emerging from lateral bipolar neurons. **f** Detail of the anterior part of the dorsal 5-HT+ neurite bundle and epidermal collar region. This part is discontinuous with the posterior part of the 5-HT neurite bundle shown in **g**, see also *double arrowheads and dashed area*. **g** The posterior part of the dorsal 5-HT+ neurite bundle contains few serotonin-LIR neurites and is discontinuous with the anterior part of the 5-HT neurite bundle, see *double arrowheads and dashed area*. **h** Partial Z- projection of a sagittal scan of the dorsal collar region. The collar cord comprises ventral neurite bundles and a dorsal sheath of somata, which are not serotonin-LIR positive. **i** Close-up of a part of the proboscis region showing the 5-HT+ bipolar neurons and the basiepidermal nervous plexus. 5-HT, serotonin; a, anus; ac-α-tub, acetylated α-tubulin; cn, circumferential neurite; cc, collar cord; ci, cilia; co, collar; dnb, dorsal neurite bundle; ep, epidermis; gs, gill slit; msp, mesocoel pore; nn, nervous plexus; onr, opisthotroch nerve ring; pnr, prebranchial nerve ring; pr, proboscis; ps, proboscis stem; sn, serotonin-LIR neuron; tb, tongue bar; tm, trunk musculature; vnb, ventral neurite bundle

After about 3-day postsettlement, most of the juveniles exhibit two pairs of gill slits (Fig. 5a, b). Both gill slits are U-shaped and heavily ciliated on the inside (Fig. 5b). No synapticles are developed. The juveniles measure up to 1.5 mm in total length (Fig. 5a–c). The proboscis shows the typical acorn shape, while the mesosome has developed into a 200-μm-long three-lobed collar region (Fig. 5a). Scanning electron micrographs and acetylated-α-tubulin-LIR reveal that the proboscis and collar region are evenly covered with cilia, whereas on the trunk region, only scattered tufts of cilia are present (Fig. 5a, c).

Throughout the epidermis of the proboscis, numerous serotonin-LIR bipolar neurons are intercalated between the other epidermal cells (Fig. 5c, i). Each soma projects a single proximal neurite into the basiepidermal plexus of the proboscis region (Fig. 5i). The most condensed area of serotonin-LIR neurites is present dorsally at the base of the proboscis, comprising a part of the proboscis plexus (Fig. 5c, d). This dense network of neurites measures approximately 200 μm in width and 100 μm in length. From here, three distinct serotonin-LIR neurite bundles leave the proboscis plexus posteriorly to pass through the subepidermal collar cord within the collar region (Fig. 5d). These serotonin-LIR neurite bundles are part of the ventral layer of neurites of the collar cord (Fig. 5h). The dorsal area of the collar cord is composed of numerous cells, presumably neurons, which do not show serotonin-LIR in any of the somata (Fig. 5h). At the posterior pole of the collar region, the three serotonin-LIR neurite bundles become basiepidermal again and connect to the nervous system within the trunk region. The median neurite bundle, composed of 7–10 serotonin-LIR neurites, continues directly into the dorsal midline and is part of the dorsal nerve cord (Fig. 5f). Serotonin-LIR is detectable until the posterior pole of the animals, with a discontinuity of about 150 μm in the middle of the trunk region (Fig. 5f, g, double arrowheads). Several serotonin-LIR bipolar neurons are located dorsolaterally close to the dorsal midline and project a short neurite into the dorsal neurite bundle (Fig. 5f). The lateral pair of neurite bundles that leaves the collar cord posteriorly runs circumferentially to the ventral side (Fig. 5d). At the level of the dorsolateral gill slits, the bilateral neurite bundles form a distinctive network around the mesocoelic pores as also described for younger stages (Fig. 5d). The bilateral neurite bundles are part of the prebranchial nerve ring and project into the ventral nerve cord. Numerous serotonin-LIR neurites extend along the entire midline of the trunk region thereby running within the ventral nerve cord that is about 40 μm wide (Fig. 5c, e). Several serotonin-LIR somata are interspersed throughout the trunk epidermis. The majority of these bipolar neurons project a proximal neurite running circumferential into the ventral nerve cord (Fig. 5e). The serotonin-LIR somata of the very posterior end send a proximal neurite running radially into the ventral neurite bundle (Fig. 5e). It should be noted that a serotonin-LIR basiepidermal plexus is only present in the epidermis of the proboscis and collar, but completely absent within the trunk region, where instead a condensed dorsal and ventral neurite bundle is present.

The analysis of serial sections for electron microscopy of the trunk epidermis in juvenile *B. misakiensis* shows comparatively few basiepidermal neurites in the epidermis (Fig. 3b, d and inset). Neurite bundles composed of 4–8 neurites are scattered throughout the lateral areas of the trunk epidermis (Fig. 3d). The most condensed neurite bundles run along the ventral and dorsal midline of the trunk region and comprise the nerve cords.

### *Saccoglossus kowalevskii*: dorsal kink stage

**Fig. 6:**
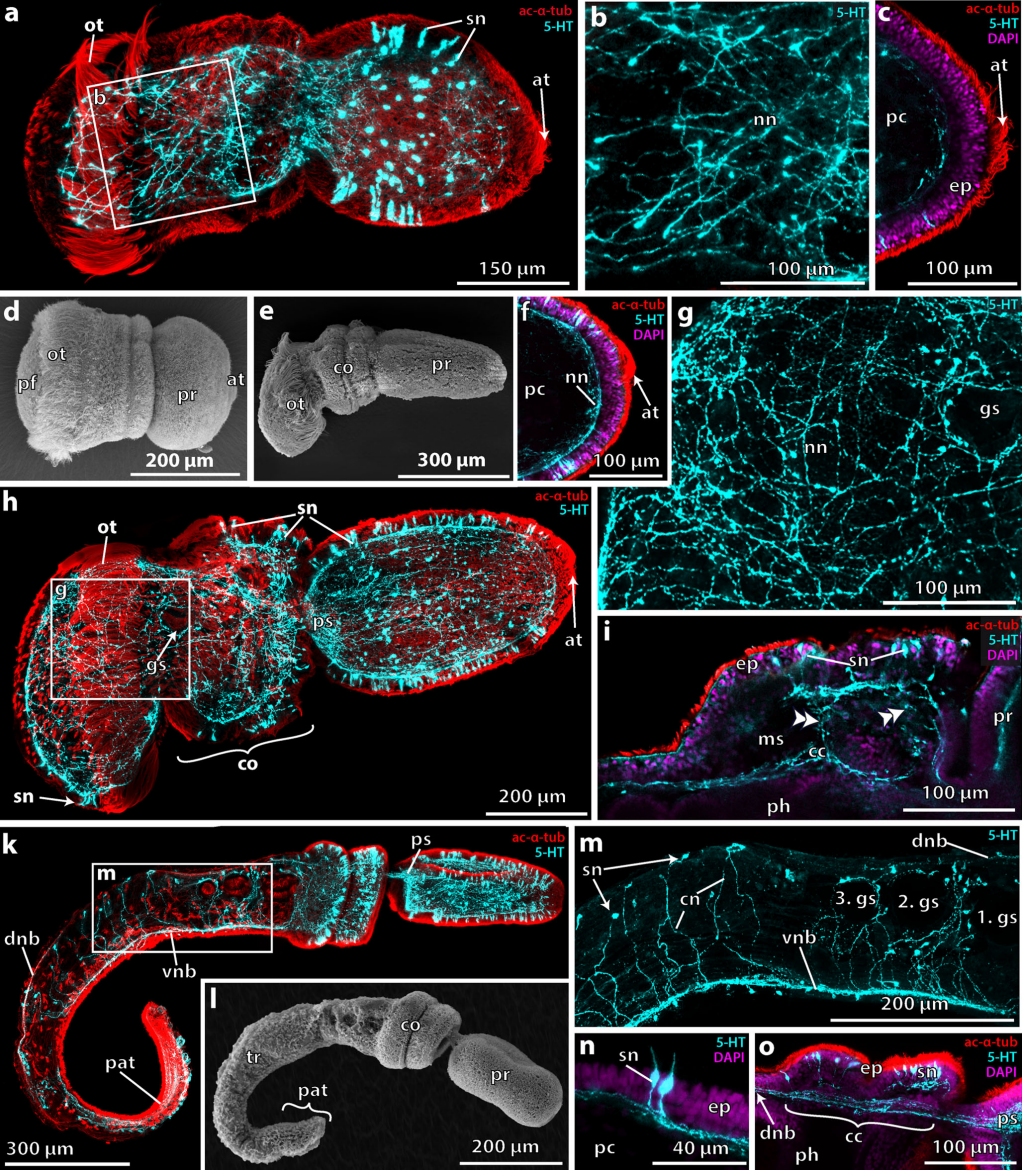
Neurogenesis in *S. kowalevskii*. **a**–**C**, **f**–**k**, **m**–**o** Z-projections of confocal microscopy image stacks. **d**, **e**, **l** Scanning electron micrographs (SEM). Anterior is to the right and ventral to the bottom in all images. **a** Overview of a dorsal kink stage. Serotonin-LIR neurons are present in the proboscis epidermis. Note the larval apical ciliary tuft. **b** Detail showing the developing nervous plexus in the trunk region of a dorsal kink stage. **c** Partial Z-projection of the anterior tip of a dorsal kink stage highlighting the absence of serotonin-LIR somata from the apical plate. **d** SEM of dorsal kink stage. **e** SEM of 1-gill-slit stage. **f** Partial Z-projection of the anterior tip of a 1-gill-slit stage. **g** Detail showing an elaborate 5-HT+ nervous plexus in the trunk region of the 1-gill-slit stage. **h** Overview of a 1-gill-slit stage showing the entire serotonin-LIR nervous system. **i** Close-up of the dorsal collar region demonstrating the 5-HT+ part in the neurulating collar cord. *Double arrowheads* point to dorsal connections of the anterior portion of the collar cord that is still in contact with the epidermis. **k** Overview of the serotonin-LIR nervous system of a 3-gill-slit juvenile. **l** SEM of a 3-gill-slit juvenile in lateral view. **m** Detail of the anterior trunk region of a 3-gill-slit juvenile showing scattered serotonin-LIR bipolar neurons projecting into the ventral neurite bundle. **n** Detail of two biploar neurons within the proboscis epidermis. **o** Detail of the collar region showing 5-HT+ neurites within the subepidermal collar cord. 5-HT, serotonin; ac-α-tub, acetylated α-tubulin; at, apical tuft; cn, circumferential neurite; cc, collar cord; co, collar; dnb, dorsal neurite bundle; ep, epidermis; gs, gill slit; ms, mesocoel; nn, nervous plexus; ot, opisthotroch; pat, postanal tail; pc, protocoel; pf, perianal field; ph, pharynx; pr, proboscis; ps, proboscis stem; sn, serotonin-LIR neuron; tr, trunk; vnb, ventral neurite bundle

Embryos at this stage are of elongate shape measuring approximately 700 μm in maximum length (Fig. 6a). The anterior proboscis region is of ovoid shape with a tapering anterior tip and is subdivided from the posterior body regions by a deep circular constriction (Fig. 6a, d). The proboscis region is covered by short cilia, whereas at the anterior tip, a ciliary tuft composed of long cilia is present (Fig. 6a, c, d). A second, yet shallow circular groove is discernable within the prospective collar region (Fig. 6d). The opisthotroch is composed of compound cilia of multiciliary cells and demarcates the posterior perianal field (Fig. 6a, d).

Serotonin-LIR reveals the presence of several bipolar neurons that are located circumferentially around the midlevel of the proboscis region (Fig. 6a). These bipolar neurons project a proximal neurite into a basiepidermal plexus of the proboscis region. A distal neurite connects to the apical cell surface where a single cilium is present. α-Tubulin-LIR reveals an apical ciliary tuft at the anterior tip; yet, no serotonin-LIR somata are detectable within the apical region close to the apical tuft (Fig. 6a, c). Throughout the prospective collar and trunk region, basiepidermal serotonin-LIR neurites form a loose network that thins out posteriorly into individual neurites within the perianal field (Fig. 6b). No traces of a serotonin-LIR opisthotroch neurite bundle are present within the trunk region, although tubulin staining reveals the existence of an opisthotroch (Fig. 6a).

### *S. kowalevskii*: 1-gill-slit hatchling

The 1-gill-slit hatchling measures between 800 μm and 1 mm in length when fully expanded (Fig. 6e, h). The animals exhibit a conical proboscis with an apical tuft composed of long cilia at the anterior tip (Fig. 6f, h). The opisthotroch is present within the trunk region, just posterior to the position of the first pair of dorsolateral gill pores (Fig. 6h). The posterior trunk region including the perianal field is elongated ventrally to a total length of 150 μm (Fig. 6e, h).

The serotonin-LIR nervous system comprises a well-developed basiepidermal plexus that extends throughout all three body regions (Fig. 6h, g). Numerous serotonin-LIR epithelial neurons are present throughout the epidermis of the proboscis. These bipolar neurons are evenly distributed except for the most anterior area around the apical ciliary tuft. Within a radius of approximately 65 μm from the central apical tuft, no serotonin-LIR somata are present (Fig. 6f, h). A higher density of serotonin-LIR neurites characterizes a part of the proboscis plexus dorsally at the base of the proboscis (Fig. 6h). Within the collar epidermis, serotonin-LIR somata are concentrated into two multi-rowed rings, encircling the anterior and middle collar region (Fig. 6h, i). The soma is located within the apical part of the epidermis, and each bipolar neuron sends a long proximal neurite into the basiepidermal plexus of the collar region. Detection of serotonin-LIR in the collar region reveals a neurite bundle that passes through the neurulating collar cord (Fig. 6i). While the posterior part is already clearly in a subepidermal position, the more anterior part shows dorsal connections to the basiepidermal plexus within the collar epidermis (Fig. 6i). The collar cord in *S. kowalevskii* neurulates successively from posterior to anterior, leaving just a neuropore at the posterior and anterior margin of the collar region (for more details, see Kaul and Stach 2010). Tubulin-LIR reveals the opisthotroch within the trunk region that propels the living hatchlings through the water (Fig. 6h). No serotonin-LIR opisthotroch neurite ring is present (Fig. 6h, g). Serotonin-LIR at the ventroposterior pole of the trunk region shows the presence of approximately 10 to 15 epithelial bipolar neurons (Fig. 6h).

**Fig. 7:**
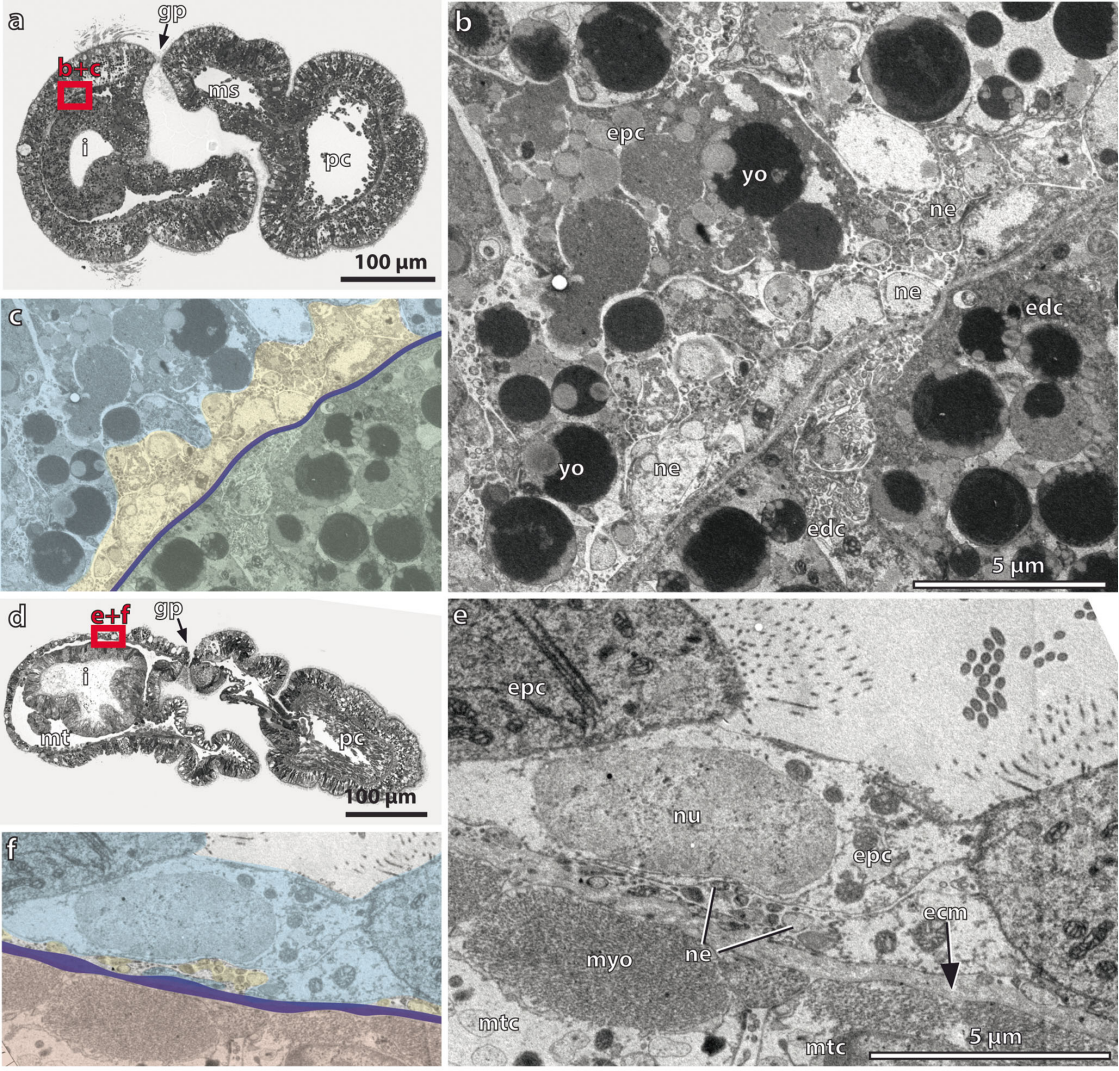
Ultrastructural details of the developing nervous system in *Saccoglossus kowalevskii*. **a** Sagittal section of a 1-gill-slit hatchling of *S. kowalevskii*. **b** Ultrastructural detail of the *red-marked box* in **a** showing a continuous layer of neurites of 2–4-μm thickness. **c** Color-coded image of the micrograph shown in **b. d** Sagittal section of a 2-gill-slit juvenile of *S. kowalevskii*. **e** Ultrastructural detail of the *red-marked box* in **c** showing only single and scattered, small neurite bundles. **f** Color-coded image of the micrograph shown in **e**. Color code for **c** and **f**: ectoderm, *light blue*; neurites, *yellow*; basal lamina (ecm), *dark blue*; endoderm, *green*; mesoderm, *pale red*. bl, blastocoel; ecm, extra cellular matrix; epc, epidermal cell; gp, gill pore; i, intestine; ms, mesocoel; mt, metacoel; mtc, metacoelic cell; myo, myofilaments; ne, neurite; nu, nucleus; pc, protocoel; yo, yolk

In transmission electron micrographs, numerous basiepidermal neurites of different diameters, exhibiting oblique to oval profiles, are present in 1-gill-slit hatchlings of *S. kowalevkii* (Fig. 7a–c). The high number of neurites composes a continuous and circumferential neurite layer of 2- to 4-μm thickness (Fig. 7b, c).

### *S. kowalevskii*: 3-gill-slit juvenile

The juveniles at this stage of development resemble the adult acorn worms in many aspects (Fig. 6k, l). Obvious differences are a lower number of gill slits and the presence of the postanal tail, a feature only present in juvenile acorn worms of the family Harrimaniidae (Cameron 2005; Stach and Kaul 2012). Scanning electron micrographs of the anterior tip of the proboscis show that the former ciliary tuft is degraded (Fig. 6k, l). The opisthotroch is partially altered and has transformed into a ventral creeping sole.

Within the proboscis and collar region, a serotonin-LIR basiepidermal plexus is present. The concentration of neurites is denser at the dorsoposterior base of the proboscis, constituting the proboscis plexus (Fig. 6k, o). Serotonin-LIR bipolar neurons are distributed throughout the epidermis of the proboscis with the exception of the most anterior region. Each soma has a bulbous central part containing the nucleus and a slender distal neurite reaching the apical cell surface, where a single cilium is present (Fig. 6n). Basally from the bulbous part, each soma sends a proximal neurite into the basiepidermal plexus. The dorsal proboscis stem sends two serotonin-LIR neurite bundles posteriorly into the subepidermal collar cord, thereby passing the proboscis neck region. At the posterior end of the collar region, the serotonin-LIR becomes superficial again and continues into a dorsal neurite bundle (Fig. 6m, o). Unlike *B. misakiensis*, *S. kowalevskii* lacks mesocoelic pores as well as a prebranchial nerve ring at this stage of development. A vast number of serotonin-LIR bipolar neurons are present within the epidermis of the anterior half of the collar region (Fig. 6k, o). The architecture of the serotonin-LIR nervous system within the trunk region differs considerably from that described for the preceding hatchling stage. Serotonin-LIR is present in a longitudinal ventral as well as dorsal neurite bundle along the trunk region (Fig. 6k, m). The ventral bundle is more extensive and extends to the posterior end of the postanal tail. The dorsal serotonin-LIR neurite bundle is less voluminous and is composed of few neurites only. It runs along the dorsal midline to the anus, with an interruption of the serotonin-LIR signal in the middle region of the trunk (Fig. 6k, m). Several serotonin-LIR bipolar neurons are present within the trunk region (Fig. 6m). The majority of the somata are located dorsolaterally, sending a proximal neurite into the ventral nerve cord (Fig. 6m). The number of serotonin-LIR somata in the trunk region is particularly low compared to the collar and proboscis region. Between the gill pores, a higher density of serotonin-LIR neurons is present. Notably, serotonin-LIR does not show a plexus-like pattern in the trunk region as in earlier stages.

Ultrastructural investigations of transmission electron micrographs of a 2-gill-slit juvenile show that only scattered neurites are present below the one-layered epidermis (Fig. 7d–f). The majority of neurites is concentrated within the dorsal and ventral nerve cord (read also Kaul and Stach 2010).

## Discussion 

### Comparative aspects of different developmental modes in enteropneusts

The nervous system of the tornaria larva features a prominent apical organ comprising a paired cluster of serotonin-LIR somata connected by a median neuropil, a ciliary apical tuft (Fig. 2b, f), as well as neurite bundles along the ciliary bands (Nielsen and Hay-Schmidt 2007; Miyamoto et al. 2010), which are particularly distinct below the opisthotroch. A similar neuronal architecture is present in the various larvae of echinoderms (Burke et al. 2006; Byrne et al. 2007). Moreover, tornariae and echinoderm larvae share further characteristics, for instance the presence of a specific ciliary band called neotroch, tricoelomate organization of the mesoderm, an excretory system involving the protocoel and a hydropore, together supporting homology of these larvae (Nielsen 2012). Tornaria and echinoderm larvae have thus been grouped together under the term “dipleurula”-larva and represent a common feature for Ambulacraria (Metschnikoff 1881). Consequently, direct development without a tornaria is supposed to have evolved within Enteropneusta. In *S. kowalevskii*, a directly developing enteropneust, no serotonin- or FMRFamid-LIR neurons are present at the apical plate during development (Fig. 6, data not shown for FMRF), nor is a serotonin-LIR opisthotroch neurite bundle detectable at any time. In contrast, a ciliary apical tuft and an opisthotroch consisting of multiciliary cells are very well present during embryogenesis in *S. kowalevskii* (Fig. 6). Serotonin- and FMRFamid-LIR bipolar receptor cells are common for most bilaterian apical organs and are supposed to be plesiomorphic for deuterostome larvae (Hay-Schmidt 2000; Marlow et al. 2014). In particular, the apical organ of echinoderm larvae is thought to function in different sensory tasks and may also play a role in metamorphosis (Byrne et al. 2007). In this respect, the absence an opisthotroch neurite bundle and of serotonin- and FMRFamid-LIR neurons from the apical organ in *S. kowalevskii* could be interpreted as a derived feature of the direct developer *S. kowalevskii*. In accordance with this interpretation, it might be concordant with the direct development and lack of metamorphosis in this species.

### Branchial nerves in deuterostomes

Early settled juveniles of *B. misakiensis* exhibit one pair of serotonin-LIR prebranchial neurites that leave the collar cord posterolaterally and run circumferentially to the ventral side, thereby forming a network around the mesocoelic pores. Mesocoelic pores are present in the pterobranch hemichordates *Rhabdopleura*, *Cephalodiscus* as well as in the majority of enteropneusts and represent a derived feature of Hemichordata (Benito and Pardos 1997). Recently, the presence of similar nerves has been documented in the pterobranch *Cephalodiscus gracilis* (serotonin- and tubulin-LIR), and homology with branchial nerves of craniates has been suggested (Stach et al. 2012). In craniates as well as *C. gracilis*, the branchial nerves curve dorsally around the pharyngeal opening and branch into an anterior pretrematic branch and a posttrematic branch (e.g., Kuratani et al. 1997). Furthermore, serotonin-LIR neurites are especially present in the pretrematic branch. In *C. gracilis*, the serotonin-LIR fibers run along the opening of the mesocoelic pores (Stach et al. 2012), i.e., corresponding to the prebranchial neurites present in *B. misakiensis*. However, in 2-gill-slit juveniles of *B. misakiensis*, only a single pair of serotonin-LIR prebranchial neurite bundles is present, and in developmental stages up to 3-gill-slit juveniles of *S. kowalevskii*, no serotonin-LIR prebranchial neurite bundle is present. Instead, several serotonin-LIR neurons are present between the gill slits of *B. misakiensis* and *S. kowalevskii*, projecting neurites into the ventral nerve cord (e.g., Fig. 6m). These neurites may correspond to a part of the branchial nerves of craniates. A more thorough characterization of the nervous system around the gill pores using other neuronal markers and probably gene expression studies are necessary to elucidate this issue further.

### Integrative centers *aka* “brains” in pterobranch and enteropneust hemichordates

According to positional congruence within the dorsal mesosome, the brain of pterobranchs and the collar cord of enteropneusts have been homologized earlier (Dilly et al. 1970; Dilly 1975; Benito and Pardos 1997). Both have been characterized as integrative centers according to ultrastructural as well as immunocytochemical features (Kaul and Stach 2010; Stach et al. 2012). The pterobranch brain is subdivided into an anterior cluster of serotonin-LIR somata and a posterior neurite plexus (Stach et al. 2012). In the present study, we could show that the collar cord of enteropneusts indeed comprises serotonin-LIR neurite bundles that are accompanied by a dorsal sheath of cells; yet, none of the dorsal collar cord somata stains positively for serotonin in any of the two species. In contrast, the entire collar epidermis contains numerous serotonin-LIR bipolar neurons. This was also shown in adult specimens of *Meioglossus psammophilus* (Worsaae et al. 2012). According to fossils of tubicolous enteropneusts (Caron et al. 2013), developmental data from pterobranchs (Stach 2013) as well as some molecular phylogenetic analyses (Cannon et al. 2009; Worsaae et al. 2012), pterobranchs might have evolved from an enteropneust-like ancestor that had a neurulated collar cord. Accordingly, pterobranchs must have reduced or lost the neurulation process of the mesosomal brain secondarily. Unfortunately, basically nothing is known about neurogenesis in pterobranchs so far, and data on the general development are also scarce (Nielsen 2012). Future studies on pterobranch development including a molecular characterization of the nervous system are needed in order to support or reject this hypothesis.

### The serotonin-LIR nervous system: of nets and cords

Our immunocytochemical data show a substantial, basiepidermal serotonin-LIR plexus in metamorphosing larvae and in early settled juveniles of *B. misakiensis.* In older juveniles, this plexus has disappeared in the trunk region, and the concentrated ventral and dorsal neurite bundles develop secondarily. In the directly developing *S. kowalevskii*, we found a similar development. It remains unclear, however, if it is the plexus itself that remodels into the concentrated neurite bundles, or if the plexus degrades and neurite bundles plus somata form de novo. In any case, a serotonin-LIR plexus precedes the formation of the serotonin-LIR dorsal and ventral neurite bundles within the trunk region in enteropneusts. The presence of a serotonin-LIR plexus was also briefly mentioned in metamorphosed *Ptychodera flava*; however, establishment of the concentrated parts, e.g., collar cord and trunk nerve cords, was not shown, because older juveniles were not investigated (Nielsen and Hay-Schmidt 2007). In order to test whether other parts of the (nonserotonin-LIR) enteropneust nervous system show a similar developmental pattern, we used TEM. The advantage of this technique is that it visualizes the factual number of all present neurites. Our TEM data show a continuous and circumferential layer of basiepidermal neurites in the trunk region in early stages of both species, whereas in juvenile worms, the majority of neurites is present within the dorsal and ventral nerve cords. These results do not contradict the assumption that the entire nervous system passes an initial plexus state prior to the formation of the concentrated cords. Actually, a similar pattern of development is seen in gene expression analyses of neural differentiation markers such as *elaV*, *bruA*, and *dclk* (Cunningham and Casey 2014) and further in studies of additional neuronal markers (Lowe et al. 2003; Nomaksteinsky et al. 2009; Pani et al. 2012). However, a recent study in another enteropneust species, *Balanoglossus simodensis*, reveals a considerable earlier formation of the nerve cords by synaptotagmin-LIR (Miyamoto et al. 2010). Surprisingly, a synaptotagmin-LIR plexus in the trunk region was not described in metamorphosing *B. simodensis*; instead, the concentrated dorsal and ventral nerve cord were already present (Miyamoto et al. 2010). This could mean that different parts of the nervous system project into the nerve cords at different time points during development and that the nerve cords in fact develop earlier as detected by serotonin-LIR in our study. However, *B. simodensis* is a different enteropneust species with a slightly different larval development than *B. misakiensis* (Urata and Yamaguchi 2004; Miyamoto and Saito 2007), and since Miyamoto and colleagues did not mention a plexus in the trunk region, it is still possible that the nervous plexus indeed develops earlier than the nerve cords.

Concluding so far, the serotonin-LIR data from this study and the ultrastructural details as well as the results from various gene expression analyses (Lowe et al. 2003; Nomaksteinsky et al. 2009; Pani et al. 2012; Cunningham and Casey 2014) allow the assumption that the enteropneust CNS passes through a transitory plexus stage. In contrast, the CNS of chordates, as well as many protostomes, develops a priori without passing a plexus or nerve net stage (Schoenwolf and Smith 1990; Wicht and Lacalli 2005; Brinkmann and Wanninger 2008; Kristof et al. 2008; Voronezhskaya et al. 2008; Wanninger 2009 and references therein; Fritsch and Richter 2010; Hindinger et al. 2013). With the numerous novel details on enteropneust neurogenesis during metamorphosis provided in this study, we show that the development of the serotonin-LIR nervous system in enteropneusts is highly conserved among species and independent from the mode of development (direct vs indirect). Because comparable datasets from developing juvenile echinoderms are only fragmentary (Burke et al. 2006; Burke 2011), yet entirely lacking in pterobranchs, it remains unclear whether passing a plexus stage during early neurogenesis could be apomorphic for Hemichordata (Pterobranchia + Enteropneusta) or even Ambulacraria (Hemichordata + Echinodermata). However, it cannot be ruled out at present that the basiepidermal plexus of hemichordates is a plesiomorphic character inherited from the LCAD that might have had both, a plexus and concentrated parts, rather than solely a complex CNS as proposed by others (Denes et al. 2007; Arendt et al. 2008; Holland et al. 2013).

The presence of serotonin-LIR neurons within the CNS of various bilaterian animals including annelids, arthropods, molluscs, cephalochordates, tunicates, and vertebrates is well established (Kristof et al. 2008; Fritsch and Richter 2010; Harzsch 2004; Candiani et al. 2001; De Bernardi et al. 2006). For instance, in adult cephalochordates, a group of serotonin-LIR interneurons is present in the roof of the cerebral vesicle (Holland and Holland 1993). Further serotonin-LIR interneurons are arranged in ventromedial parallel chains along the spinal cord (Candiani et al. 2001). In ascidian larvae, most serotonin-LIR compartments are confined to the brain, closely positioned to the sensory vesicle (Stach 2005; De Bernardi et al. 2006). Additionally, serotonin-LIR receptor neurons are present within the epidermis of the tail as well as within the adhesive papillae and are part of the peripheral nervous system (PNS) (De Bernardi et al. 2006). In vertebrates such as teleosts, amphibians, reptiles, or mammals, one to two clusters of serotonin-LIR interneurons are present at the level of the otic placodes with posterior directed projections into the spinal cord (Ekström et al. 1985; Van Mier et al. 1986; Wallace 1985; Wallace and Lauder 1983). Interestingly, all serotonin-LIR neurons that could be detected in *B. misakiensis* and *S. kowalevskii* are epithelial, bipolar neurons with a distal neurite reaching the apical surface where a cilium is present. Each soma is intercalated between the other epidermal cells and a proximal neurite projects either into the basiepidermal plexus or into one of the concentrated nerve cords. Such a specific neuronal morphology leads to assume that the bipolar neurons are ciliary receptor cells that are involved in uptake of sensory stimuli (Richter et al. 2010). Thus, no interneurons could be identified with serotonin-LIR in *B. misakiensis* and *S. kowalevskii*. Consistent with numerous similarities in the anteroposterior and mediolateral architecture including position of specific neuronal types (e.g., serotonin-LIR interneurons) between the ventral CNS of some protostomes (annelids and arthropods) and the dorsal CNS of chordates, homology of serotonin-LIR interneurons has been proposed by some authors (Denes et al. 2007; Arendt et al. 2008; Holland et al. 2013). The absence of such serotonin-LIR interneurons in the enteropneust nervous system can thus be regarded as secondary.
